# RIP140 Represses Intestinal Paneth Cell Differentiation and Interplays with SOX9 Signaling in Colorectal Cancer

**DOI:** 10.3390/cancers13133192

**Published:** 2021-06-26

**Authors:** Antoine Gleizes, Mouna Triki, Sandrine Bonnet, Naomi Baccari, Gabriel Jimenez-Dominguez, Aurélie Covinhes, Nelly Pirot, Philippe Blache, Rong Yuan, Balázs Győrffy, Vincent Cavaillès, Marion Lapierre

**Affiliations:** 1 IRCM—Institut de Recherche en Cancérologie de Montpellier, INSERM U1194, Université de Montpellier, Institut Régional du Cancer de Montpellier, CNRS, 208 rue des Apothicaires, F-34298 Montpellier, France; antoine.gleizes@inserm.fr (A.G.); Mouna.TRIKI@lyon.unicancer.fr (M.T.); sandrine.bonnet@inserm.fr (S.B.); naomi.baccari@etu.umontpellier.fr (N.B.); gabriel.jimenez-dominguez@etu.umontpellier.fr (G.J.-D.); philippe.blache@inserm.fr (P.B.); vincent.cavailles@inserm.fr (V.C.); 2 BioCampus, RHEM, Université de Montpellier, CNRS, INSERM, F-34093 Montpellier, France; aurelie.covinhes@inserm.fr (A.C.); nelly.pirot@inserm.fr (N.P.); 3 Department of Medical Microbiology, Immunology and Cell Biology, School of Medicine, Southern Illinois University, Springfield, IL 62794-9628, USA; ryuan@siumed.edu; 4 Department of Bioinformatics, Semmelweis University, 1094 Budapest, Hungary; gyorffy.balazs@med.semmelweis-univ.hu; 5 Lendület Cancer Biomarker Research Group, Research Centre for Natural Sciences, 1117 Budapest, Hungary

**Keywords:** intestinal epithelium, cell differentiation, colon cancer, RIP140, SOX9, transcriptional regulation

## Abstract

**Simple Summary:**

In the small intestine, the transcription factor SOX9 regulates the differentiation of Paneth cells, which are key actors in the intestinal antimicrobial defense and stem cell niche. SOX9 is also an important player in colon cancer development and progression. In this study, we demonstrate that the transcription coregulator RIP140 inhibits SOX9 expression and activity. Consequently, RIP140 and SOX9 exert opposite effects on Paneth cell differentiation and colon cancer cell proliferation. Their expression undergoes inverse regulation by the Wnt signaling pathway and inversely correlates with survival of patient with colon cancer. These results identify RIP140 as a major regulator of SOX9 signaling with functional relevance in intestinal physiopathology.

**Abstract:**

RIP140 is a major transcriptional coregulator of gut homeostasis and tumorigenesis through the regulation of Wnt/APC signaling. Here, we investigated the effect of RIP140 on Paneth cell differentiation and its interplay with the transcription factor SOX9. Using loss of function mouse models, human colon cancer cells, and tumor microarray data sets we evaluated the role of RIP140 in SOX9 expression and activity using RT-qPCR, immunohistochemistry, luciferase reporter assays, and GST-pull down. We first evidence that RIP140 strongly represses the Paneth cell lineage in the intestinal epithelium cells by inhibiting Sox9 expression. We then demonstrate that RIP140 interacts with SOX9 and inhibits its transcriptional activity. Our results reveal that the Wnt signaling pathway exerts an opposite regulation on SOX9 and RIP140. Finally, the levels of expression of RIP140 and SOX9 exhibit a reverse response and prognosis value in human colorectal cancer biopsies. This work highlights an intimate transcriptional cross-talk between RIP140 and SOX9 in intestinal physiopathology.

## 1. Introduction

The intestines are one of the most rapidly self-renewing tissues. Due to permanent exposure to external compounds, the intestinal epithelium needs to be constantly renewed to preserve its integrity and homeostasis. The intestinal epithelium is a mono-stratified cellular layer shaped by developmental cues into villi and Lieberkühn crypts, and housing intestinal stem cells (ISC) at the origin of the intestinal physiology. Lgr5-expressing crypt-based columnar (CBC) cells, located at the bottom of the crypts, are constantly cycling to produce progenitor cells, sustaining the high epithelial turn-over. Progenitors divide through the transit-amplifying compartment and their progeny, constrained by proliferative pressure, migrate to the top of the villi while undergoing differentiation to become highly specialized epithelial cells [1].

Absorptive progenitors differentiate into absorptive enterocytes, the most common population that are responsible for nutrient absorption, and secretory progenitors give rise to four different specialized cell types [2]. The goblet cells secrete mucus to protect the epithelium and lubricate the passage of the alimentary bowl towards the digestive tract [3]. Enteroendocrine cells produce hormones involved in metabolism regulation, nutrient uptake, and appetite [4]. Tuft cells, which present chemosensitive properties, are mainly involved in immune response [5]. Last but not least, considered the maestro of intestinal physiology, the Paneth cells (PC) coordinate host–microbiota interactions [6], are a sensor of environmental changes [7], the source of intestinal inflammation [8], and sustain the stem cell compartment [9].

PC fate is determined by several transcription factors, which regulate the HMG-box transcription factor SOX9 involved in final PC differentiation [10,11,12]. In addition to autonomous cellular signals, which maintain CBC identity and behavior, PCs provide the membrane and soluble factors composing the so-called stem cell niche localized at the bottom of the crypts. Several major signaling pathways, including Wnt, Notch, EGF, and BMP, control intestinal crypt physiology [13,14,15,16]. The Wnt pathway is of particular interest as it regulates ISC behavior and identity, and also promotes the formation of PC through the positive regulation of *SOX9* gene expression [9,17,18].

Colorectal carcinogenesis is a multifactorial process, and among sporadic colorectal cancers (CRC), a common sequence of molecular events (the so-called adenoma-carcinoma sequence) trigger and sustain the epithelial transformation. The mutation of the gene encoding the tumor suppressor APC is a major genetic hit driving the colorectal carcinogenesis found in approximately 80% of sporadic CRC cases [19,20]. As a consequence of this mutation, epithelial cells present a constitutive β-catenin activation, which is the major effector of the Wnt pathway, and thus a strong transcriptional activation of the β-catenin–TCF complex [21]. The constitutive activation of the Wnt pathway enhances cellular stemness and has been linked to chromosomal instability, as well as other cancer promoting factors [22]. Overexpression of some Wnt pathway target genes reinforces colorectal carcinogenesis, as is the case for SOX9. Indeed, this transcription factor is over-expressed in many cancer types, including CRC [23]. SOX9 particularly promotes CRC cell proliferation and enhances tumorigenicity in vivo [23,24].

For many years, we have been deciphering the role of the Receptor-Interacting Protein of 140 kDa (RIP140), also known as the Nuclear Receptor-Interacting Protein 1 (NRIP1), which was initially identified in human cancer cells as a corepressor of the estrogen receptor α [25]. It consists of 1158 amino acids in human, and 1161 amino acids in mouse, with 83% homology between the two sequences [26]. Knowledge of the biological functions of RIP140 came from studies on genetically modified mouse models of RIP140, i.e., the RIP140 knockout (RIPKO)(for a review, see [27]). Moreover, this transcriptional coregulator is involved in many physio-pathological processes [27] and molecular networks [28]. By its ability to recruit histone deacetylases or C-terminal binding proteins, RIP140 mainly acts as a transcriptional co-repressor [29]. Our work shed light on its ability to control intestinal homeostasis and tumorigenesis through the repression of Wnt/β-catenin signaling activity by positively regulating APC expression in a transgenic mice model and colorectal cancer cell lines. In correlation with these observations, RIP140 expression is associated with a decrease in CRC cell tumorigenicity when grafted onto nude mice [30].

To better understand its role in intestinal physio-pathological processes, we further deciphered the RIP140′ cross-talk with the Wnt/SOX9 signaling pathway. Our data indicate that RIP140 inhibits SOX9 expression and transcriptional activity. Interestingly, in human CRC cells, the Wnt signaling exerts an opposite regulation on SOX9 and RIP140 gene expression, thus explaining their reverse misregulation in human CRC biopsies. Finally, RIP140 and SOX9 exhibit opposite effects on CRC cell proliferation, and their expression in human CRC biopsies conversely correlates with patient overall survival.

## 2. Materials and Methods

### 2.1. Animals

To generate the C57BL/6J mice line with conditional KO of the *Rip140* gene in the intestinal epithelium, RipKO^Int^ transgenic mice (obtained from the Yuan’s lab, Springfield, USA [31]) were crossed with mice bearing a tamoxifen-dependent Cre recombinase, expressed under the control of the villin promoter [32]. Animals were genotyped by PCR using ComR1 primers specific to the floxed region (see Table S1 for primer sequences). All animals were maintained under standard conditions, on a 12:12-h light/dark schedule and fed a chow diet ad libitum, according to European Union guidelines for use of laboratory animals. In vivo experiments were performed in compliance with the French guidelines for experimental animal studies (agreement D3417227).

### 2.2. Organoids

Ex vivo organoid culture was established with whole crypts freshly isolated from mouse intestinal tissues (adapted from [33]). After a 30 min incubation of the minced tissue with 3 mM EDTA at 4 °C, the crypts were pelleted and passed through a 70 µm strainer. Crypts were seeded in Matrigel 1:1 IntestiCult^TM^ organoid growth medium (StemCell) and maintained in IntestiCult^TM^ medium for 5 days before passaging. Organoid development was followed day by day with classic optical microscopy and pictures were taken at a 4, 10, and 20× magnification with a ZEISS Primovert microscope in brightfield. Organoids were pelleted at day 5 for RNA analysis.

### 2.3. Real-Time Quantitative PCR (RT-qPCR)

Total RNA was extracted from cells or mouse tissues using a High Pure RNA Isolation kit (Roche Applied Science) according to the manufacturer’s instructions. Sequential isolation of wild-type mouse small intestinal epithelial cells was performed as described [34]. Total RNA (1 µg) was subjected to reverse-transcription using Superscript II reverse transcriptase (Invitrogen). RT-QPCR was performed on a Light Cycler 480 SYBR Green I Master (Roche Applied Science) and was carried out in a final volume of 10 μL using 0.25 μL of each primer (25 μM), 5 μL of the supplied enzyme mix, 2.5 μL of H_2_O, and 2 μL of the template diluted at 1:10. After pre-incubation at 95 °C, runs corresponded to 35 cycles of 15 s each at 95 °C, 5 s at 60 °C, and 15 s at 72 °C. Melting curves of the PCR products were analyzed using the Light Cycler software system to exclude amplification of unspecific products. Results were normalized to RS9 or 28S housekeeping gene transcripts. See Supplemental Table S1 for primer sequences.

### 2.4. Histological and Immunostaining Analysis

Mouse tissues were fixed with 4% paraformaldehyde, embedded in paraffin, and sectioned (3 μm). Immunohistochemistry (IHC) was performed using a VENTANA Discovery Ultra automated staining instrument (Ventana Medical Systems, Tuscon, AZ, USA) and VENTANA reagents, according to the manufacturer’s instructions. Sections were then incubated with antibodies specific for SOX9 (Ab185966, Abcam) and were counterstained with haematoxylin for 4 min and dehydrated before coverslip addition. Histology and IHC slides were scanned with a Nanozoomer Hamamatsu device (Hamamatsu Photonics, Tokyo, Japan) and analyzed with Nanozoomer Digital Pathology (NDPview2) software (Hamamatsu Photonics).

### 2.5. Cell Culture and Transfections

HCT116 were grown in McCoy medium supplemented with 10% FCS, 100 U/mL penicillin, 100 mg/mL streptomycin, and 100 mg/mL sodium pyruvate. SW480 and RKO cells were grown in RPMI medium supplemented with 10% FCS, 100 U/mL penicillin, 100 mg/mL streptomycin, and 100 mg/mL sodium pyruvate. RKO cells were transitorily transfected with small interference RNA directed to RIP140 or SOX9 (Dharmacon). All transfections were carried out using Lipofectamine2000 (Invitrogen), as recommended by the manufacturer.

### 2.6. GST Pull-Down Assay

Briefly, ^35^S-labelled proteins were cell-free-synthesized using a TNT lysate system (Promega) and incubated with purified GST fusion proteins overnight at 4 °C in NETN buffer containing 0.5% NP-40, 1 mM EDTA, 20 mM Tris pH 8, 100 mM NaCl, 10 mM dithiothreitol, and protease inhibitors (Roche Diagnostics, Meylan, France). Protein interactions were analyzed by SDS–PAGE followed by quantification using a Phosphorimager (Fujix BAS1000). Gels were stained with Coomassie Brilliant Blue (BioRad) to visualize the GST fusion proteins present in each track.

### 2.7. DuoLink Proximity Ligation Assay

The proximity ligation assay was performed to visualize interactions using a Duolink^®^ kit (Sigma-Aldrich^®^), according to the manufacturer instructions. SW480 cells were plated on slides (5 × 10^4^ cells per well) 24 h prior to fixation with paraformaldehyde 3.7% and permeabilization with Triton X-100 1%. After blocking with BSA 1% for at least 3 h, cells were incubated with two primary antibodies, RIP140 (Ab42126, Abcam) and SOX9 (Ab185966, Abcam), overnight at 4 °C. A pair of oligonucleotide-labeled secondary rabbit and goat antibodies IgG (Duolink^®^ In Situ PLA^®^ Probes) were used according to the manufacturer’s instructions to bind to the primary antibodies. This pair of secondary antibodies generate a signal only when the two probes are in close proximity (40 nm). The PLA signals were assigned using Duolink^®^ In Situ Detection Reagents Orange (554 nm laser line). Slides were counterstained with Hoechst (1/1000, Sigma Aldrich^®^) and mounted with Mowiol (Sigma-Aldrich^®^) for fluorescence microscopy. The images were obtained with ×40 magnification using an AxioImager microscope (Zeiss).

### 2.8. DNA Microarray and RNA Sequencing Analysis

Published DNA microarray datasets, GSE44076 (https://www.colonomics.org (accessed on January 2021)) and GSE39582 [35], were reanalyzed for RIP140 and SOX9 expression using the CANCERTOOL database [36]. RNA sequencing data from the TCGA [37] were reanalyzed using Cox proportional hazard regression [38], and RNAseq data obtained from CRC samples from the TCGA dataset were analyzed, as described previously [39]. The Kaplan–Meier method was used to estimate overall survival (OS) calculated from the diagnosis until death. Patients lost at follow-up were censored at the time of last contact.

### 2.9. Statistical Analysis

Data were presented as means ± SEM. Statistical comparisons were performed with Mann–Whitney or Spearman tests. Differences were considered statistically significant at *p* < 0.05. (* *p* < 0.05; ** *p* < 0.01, and *** *p* < 0.001). The log-rank test was used to test the difference between groups. Differences were considered statistically significant at a *p* < 0.05 level. STATA statistical software (STATA, College Station, TX) was used for all analyses. For the CANCERTOOL database analysis, a Student T-test (when the comparison in between two groups) or an ANOVA test (for more than two groups) was performed in order to compare the mean between groups.

## 3. Results

### 3.1. Rip140 Inhibits Sox9 Expression and Paneth Cell Lineage in Mouse Intestine

**Figure 1:**
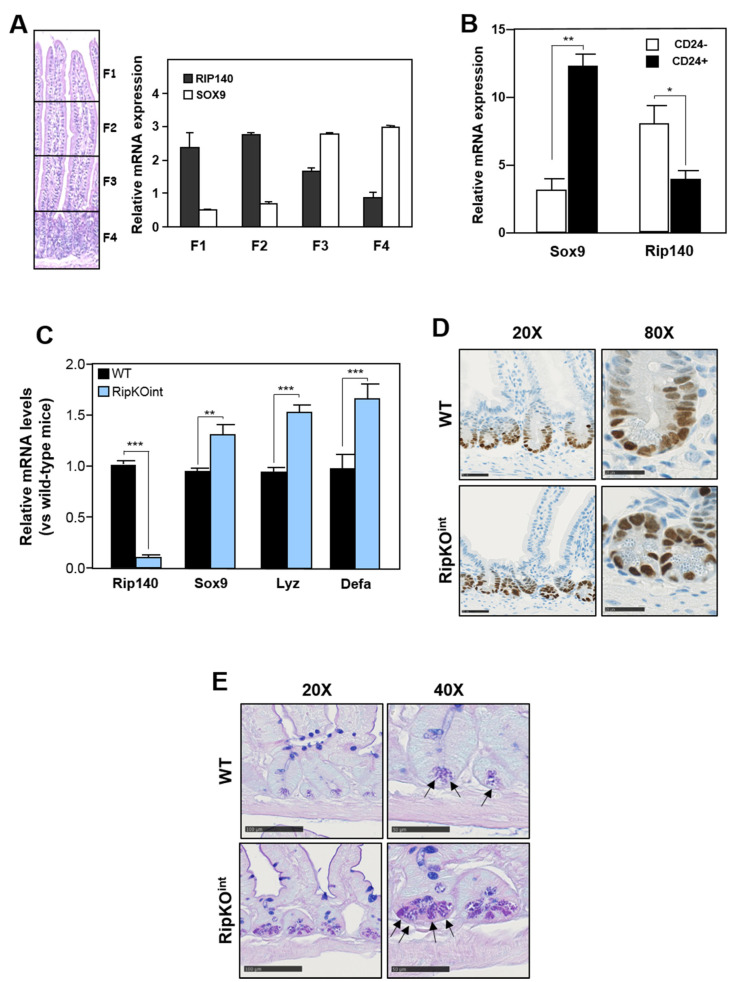
(**A**) RT-qPCR analysis of Rip140 and Sox9 in fractions of the epithelial cells along the villus—crypt axis of wild-type (WT) mouse tissues. The results are expressed in arbitrary units after normalization by RS9 mRNA levels. Values are the means ± SEM of 4 independent experiments. (**B**) Reanalysis of Rip140 and Sox9 mRNA expression from RNA sequencing of Paneth cells (CD24^+^ cells) and non-Paneth cells provided by Yu et al. [2]. (**C**) RT-qPCR analysis of Rip140, Sox9, Lysozyme (Lyz), and Defensin (Defa) in small intestines of WT and RipKO^int^ mice. (**D**) Immunostaining of Sox9 and (**E**) PAS-BA coloration of WT and RipKO^int^ small intestines. A Student t test was used for statistical analysis: * *p* < 0.05, ** *p* < 0.01 and *** *p* < 0.001.

We previously demonstrated that RIP140 was expressed in all intestinal epithelial cells with an increasing gradient along the crypt—villus axis [30]. Moreover, in constitutive Rip140 knock-out mice, Rip140 deletion was associated with an increase in the number of PC per crypt, suggesting that this transcription coregulator may drive the PC differentiation program of intestinal cells. Upon sequential isolation of wild-type mouse small intestinal epithelial cells, we observed that the levels of Rip140 mRNA were inversely correlated to those of Sox9, the master transcription factor required for PC terminal differentiation (Figure 1A). Reanalysis of data from the RNA sequencing of PC (CD24^+^ cells) and non-PC provided by Yu et al. [40] confirmed this inverse correlation between Sox9 and Rip140 expression, since high levels of Sox9 were associated with low levels of Rip140 in CD24^+^ cells and inversely in CD24^−^ ones (Figure 1B).

We next used transgenic mice exhibiting a tamoxifen-inducible specific invalidation of the Rip140 gene in the intestinal epithelium (RipKO^int^). As shown in Figure 1C, a strong decrease of Rip140 gene expression in the intestine was validated by RTqPCR, and we also confirmed that *Rip140* gene silencing in epithelial cells enhanced cell proliferation and apoptosis (data not shown). As expected, the Sox9 mRNA was increased in the RipKO^int^ mice compared to their wild-type littermates, as well as the PC markers Lyzozyme (Lyz) and Defensins (Defa; Figure 1C). By contrast, the expression of the transcription factors Math1 and Gfi1, both involved in PC differentiation, was not significantly affected (Supplemental Figure S1A). These dysregulations were confirmed in two other mouse models, namely RIPKO and RIPTg mice, with a constitutive knock-out or overexpression of the Rip140 gene, respectively (Supplemental Figure S1B). In addition, the differences observed in the gene expression levels of Sox9 and Lyz genes among the abovementioned genotypes were also correlated with their protein level by immunohistochemistry (Figure 1D,E, respectively, and Supplemental Figure S1C,D). The number of PC at the bottom of the RipKO^int^ crypts was clearly enhanced (arrows), although the higher Lyz staining intensity could also be due to more productive PC (Figure 1E).

### 3.2. Repression of Sox9 Expression and Paneth Cell Differentiation by Rip140 in Organoid Culture

**Figure 2:**
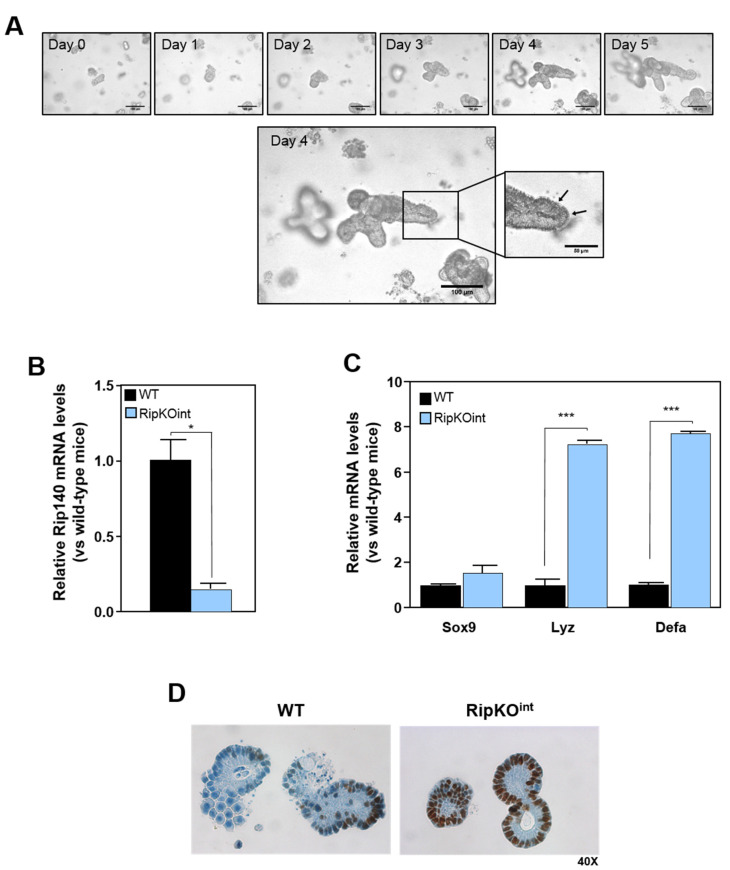
(**A**) Time course of an isolated single crypt growth. Differential phase contrast image reveals granule-containing Paneth cells at crypt bottoms (arrows). (**B**) RT-qPCR analysis of Rip140 and (**C**) Sox9, Lyz, and Defa in organoids derived from WT and RipKO^int^ mice. The results are expressed in arbitrary units after normalization by RS9 mRNA levels. Values are the means ± SEM of 3 independent experiments. (**D**) Immunostaining of Sox9 in WT and RipKO^int^ organoids. A Student t test was used for statistical analysis: * *p* < 0.05 and *** *p* < 0.001.

We next determined whether Rip140 knock-out could affect the development of intestinal epithelium ex vivo. For this, we cultured small intestinal organoids from RipKO^int^ and control mice. We performed kinetics to measure the growth efficiency and ability to form budding organoids (Figure 2A). We observed that the development of organoids was optimal at day 4, with the presence of PC in crypt-like structures. In these conditions, we confirmed by RT-qPCR the decrease of Rip140 expression in organoids from RipKO^int^ mice compared to their wild-type littermates (Figure 2B), which was again correlated with a slight increased expression of Sox9 and a significant upregulation of Lyz and Defa genes in RipKO^int^ organoids (Figure 2C). We also observed that RipKO^int^ mice exhibited a significantly increased number of Sox9-positive cells compared to wild-type animals (Figure 2D), confirming that Rip140 regulates Sox9 expression at the protein level and PC differentiation.

### 3.3. RIP140 Is Transcriptionally Repressed by the Wnt Pathway in Human Colorectal Cell Lines

**Figure 3:**
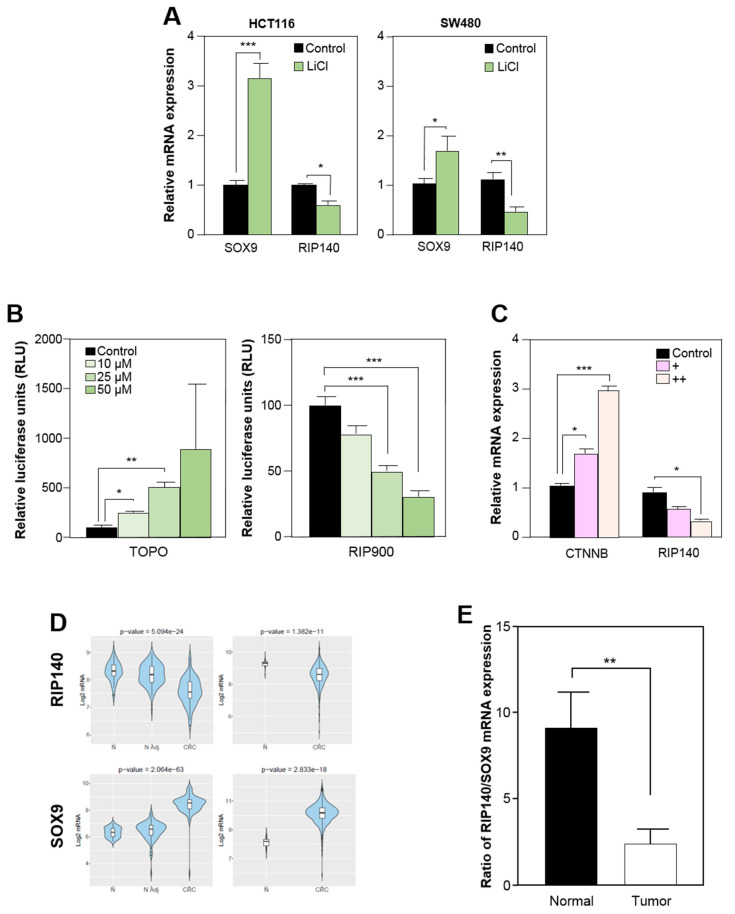
(**A**) RT-qPCR analysis of RIP140 and SOX9 expression in HCT116 (left panel) and SW480 (right panel) CRC cells after LiCl treatment (50 µM) for 24 h. The results are expressed in arbitrary units after normalization by 28S mRNA levels. Values are the means ± SEM of 3 independent experiments. (**B**) HCT116 cells were transfected with a β-catenin responsive reporter plasmid (left panel) or with RIP140 gene promoter reporter vector (right panel) and treated with a dose-response of LiCl. Relative luciferase activity was expressed as the mean ± SEM; n = 3. (**C**) RT-qPCR analysis of RIP140 and CTNNB expression after ectopic expression of TCF and β-catenin. The results are expressed in arbitrary units after normalization by 28S mRNA levels. Values are the means ± SEM of 3 independent experiments. (**D**) Analysis of RIP140 and SOX9 mRNAs expression in non-tumoral (N), CRC-adjacent non-tumoral tissues (N Adj), and CRC specimens in two different cohorts (GSE44076 on the left panel and GSE39582 on the right panel) using the CANCERTOOL database. Data are presented as violin plots showing the expression of the gene of interest (*p*-values are indicated). The *Y*-axis represents the Log2-normalized gene expression. (**E**) RIP140 and SOX9 mRNA levels expressed in arbitrary units (AU) after normalization to actin mRNA levels in 24 matched normal and tumor colon samples (means ± SEM). A Student t test was used for statistical analysis: * *p* < 0.05, ** *p* < 0.01 and *** *p* < 0.001.

Given the critical role played by the Wnt signaling pathway in SOX9 expression and PC differentiation, we sought to establish whether RIP140 expression was impacted by Wnt signaling. To demonstrate the regulation of RIP140 by the Wnt pathway, we treated HCT116 and SW480 CRC cells with LiCl to inhibit the GSK3B kinase and activate Wnt signaling. In these conditions, we observed that the expression of SOX9 was significantly increased upon Wnt activation. On the contrary and as expected, the expression of RIP140 was dramatically decreased compared to non-treated cells (Figure 3A).

We then performed a classical TCF reporter assay by transiently transfecting the two reporter plasmids (pTOPflash or pFOPflash) or the RIP900 reporter construct containing the 900 bp of the RIP140 promoter fused to the luciferase coding sequence into the HCT116 cell line in the presence of LiCl. As shown in Figure 3B, the level of β-catenin-driven transcriptional activity measured as the TOP/FOP luciferase reporter ratio was significantly increased by LiCl, in a dose-dependent manner. In contrast, we observed a clear decrease of luciferase activity upon co-transfection with a RIP140 promoter vector. This indicates that activation of the Wnt pathway was able to repress the promoter of the *RIP140* gene. Similar results were obtained upon ectopic expression of TCF and b-catenin (Figure 3C).

Owing to the major role of RIP140 and SOX9 in colon tumorigenesis, we then investigated the expression patterns of RIP140 and SOX9 in human CRC samples compared to normal colon tissues. Using the CANCERTOOL database [36], we first reanalyzed the published Affymetrix DNA microarray data from two cohorts, namely GSE44076 (https://www.colonomics.org (accessed on January 2021)) and GSE39582 [35]. As shown in Figure 3D, and in perfect agreement with the data from the mice, the results clearly showed a significant decrease of RIP140 mRNA levels in CRC biopsies, and inversely correlated with those of SOX9. We confirmed these findings using another database (TNMplot) and by comparing paired tumor and adjacent normal tissue (data not shown). These observations were also supported by RT-qPCR analysis on 24 pairs of normal and tumor colon tissue samples, showing a significant decrease of RIP140/SOX9 expression ratio in the tumoral tissues (Figure 3E).

### 3.4. RIP140 Interacts with SOX9 and Represses Its Activity

**Figure 4:**
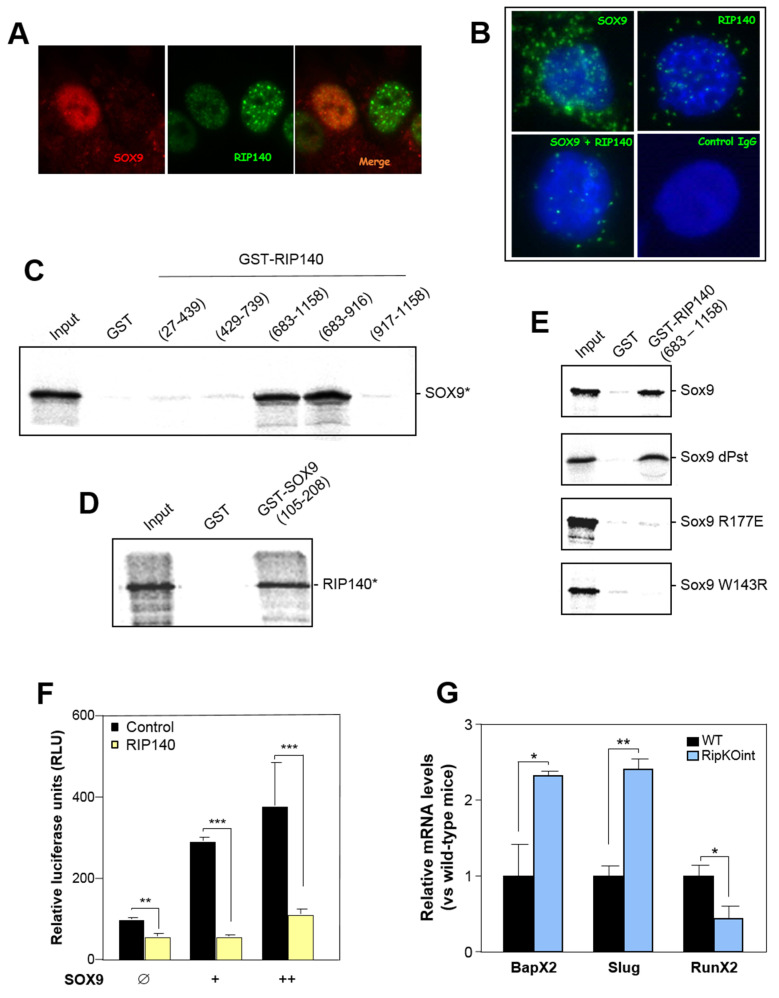
(**A**) Immunofluorescence of SOX9 and RIP140 in SW480 cells. Images show merged SOX9 (red) and RIP140 (green) detection. (**B**) In situ proximity ligation assays of SOX9 and RIP140, alone or in combination, in SW480 cells using primary anti-RIP140 and anti-SOX9 antibodies diluted in PBS–1%BSA. (**C**) GST pull-down assays were carried out using bacterially expressed GST, and GST-fused RIP140 fragments and ^35^S-labeled SOX9. (**D**) Same as in (**C**) with GST-fused SOX9 fragment and ^35^S-labeled RIP140. (**E**) GST pull-down assays were carried out as in (**C**) with GST and GST-fused SOX9 wild-type or mutant fragment and ^35^S-labeled RIP140. (**F**) HCT116 cells were transfected with SOX9 response element reporter vector together or not with RIP140 expression plasmid and increasing doses of SOX9 expression vector. Relative luciferase activity was expressed as the mean ± S.D.; n = 3. (**G**) RT-qPCR analysis of Bapx2, Slug, and Runx2 in small intestines of WT and RipKO^int^ mice. A Student t test was used for statistical analysis: * *p* < 0.05, ** *p* < 0.01, and *** *p* < 0.001.

To further characterize the effect of RIP140 on SOX9, we investigated whether the two proteins were able to colocalize and to physically interact. Using SW480 cells, we observed by immunofluorescence that SOX9 (in red) and RIP140 (in green) formed nuclear foci (Figure 4A). Moreover, when the two channels were merged, the two images could be superimposed, indicating that RIP140 and SOX9 colocalized. Interestingly, we clearly observed that cells with high levels of SOX9 corresponded to cells with low RIP140 (left cell), and conversely, cells with low SOX9 corresponded to cells with high levels of RIP140 protein (right cell). The interaction between the two endogenous proteins was also detected in proximity ligation assay in intact SW480 CRC cells (Figure 4B). Moreover, as shown in Figure 4C, GST pull-down experiments clearly demonstrated that the C-terminal region of RIP140 (fragment which encompasses the RIP140 sequence from amino acid 683 to 916) was able to bind to the in vitro translated SOX9 protein. We then performed GST pull-down experiments in order to identify which domains were involved in this interaction. Deletion mutants of SOX9 protein revealed that the C-terminal domains of RIP140 interacted with the N-terminal region (105–208) of SOX9 (Figure 4D). Interestingly, mutants for the high-mobility group (HMG) domain of SOX9 (Supplemental Figure S2A) were unable to interact with RIP140 (Figure 4E). To determine if the transcriptional activity of SOX9 was affected by this interaction, we used a luciferase reporter plasmid containing SOX9-response elements. We observed that RIP140 was able to inhibit transactivation by SOX9 (Figure 4F and Supplemental Figure S2B) and did not affect transactivation by the SOX9 mutant (Supplemental Figure S2C). To confirm the inhibition of SOX9 transcriptional activity by RIP140, we performed RT-qPCR to quantify SOX9 target genes in our mouse models and observed a significant increase of Bapx2 and Slug expression, associated with a decrease of Runx2 expression in the RipKO^int^ mice as compared to the wild-type mice (Figure 4G). Altogether, these data indicated that, in addition to the regulation of SOX9 expression, RIP140 interacted with SOX9 and inhibited its transcriptional activity.

### 3.5. RIP140 and SOX9 Are Opposite Prognosis Markers in CRC

**Figure 5:**
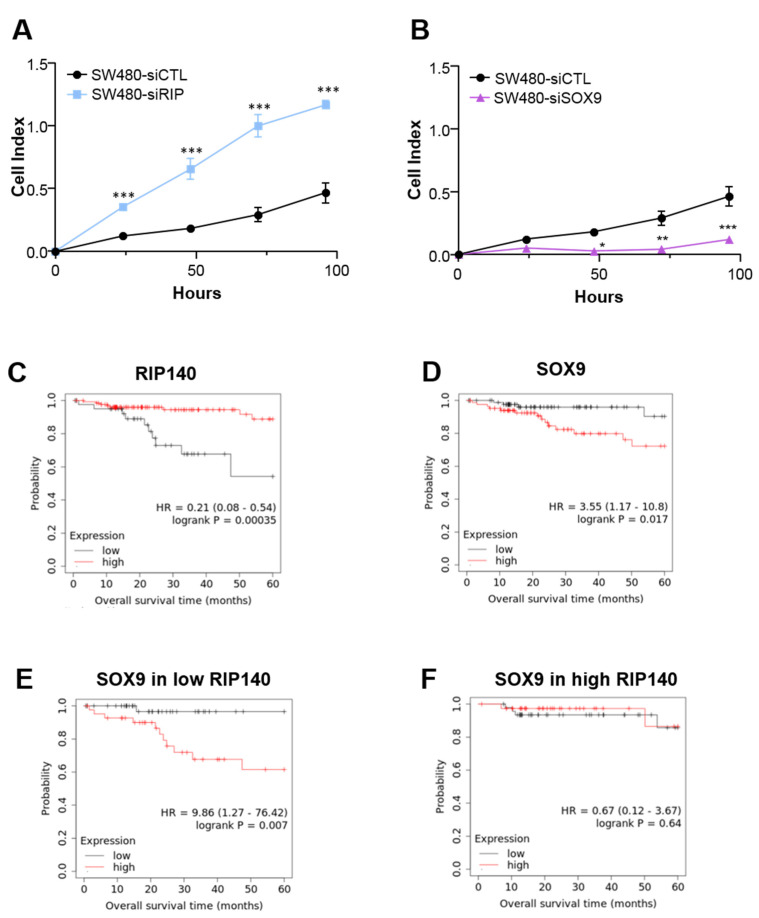
Cell index corresponding to the number of SW480 CRC viable cells, without (SW480 -control) or with siRNA-mediated knock-down of (**A**) RIP140 (SW480 -siRIP) or (**B**) SOX9 expression (SW480-siSOX9) A Student t test was used for statistical analysis: * *p* < 0.05, ** *p* < 0.01, and *** *p* < 0.001. (**C**,**D**) Kaplan-Meier analysis performed on TCGA RNA-seq data using the Kaplan–Meier plotter database. Patients were ranked according to RIP140 (**C**) or SOX9 (**D**) gene expression in their tumors and divided into two groups exhibiting low and high expression, respectively (best cut-off threshold). (**E**,**F**) Kaplan-Meier analysis of the cumulative OS of patients with low or high SOX9 gene expression (same cut-off used in panel (**D**) was performed on the groups exhibiting low or high RIP140 gene expression. A log-rank test was used for statistical analysis.

To determine the biological relevance of these regulations in human pathology, we investigated the impact of RIP140 on SOX9 oncogenicity. As shown in Figure 5A, RIP140 exerted a clear antiproliferative activity in SW480 CRC cells since its knockdown in human RKO CRC cells produced a significant mitogenic effect, confirming our previous results [30]. Conversely, silencing of SOX9 led to an inhibition of cell proliferation (Figure 5B). These data were confirmed in another cell line, RKO cells (Supplemental Figure S3A,B). Moreover, we performed rescue assays that revealed that RIP140 inhibition could reverse the inhibitory effect of SOX9 silencing on proliferation (Supplemental Figure S3C).

Based on these opposite effects on cell proliferation and on the inhibition of SOX9 signaling by RIP140, we investigated how the two transcription factors predicted the overall survival (OS) of patients with CRC. Using the Kaplan-Meier Plotter database, we reanalyzed RNAseq data obtained from 173 patients with localized colon adenocarcinoma (i.e., without lymph node invasion). We found that patients with high RIP140 mRNA levels in the tumor presented better rates of OS than patients with low RIP140 mRNA levels (Figure 5C). In contrast, SOX9 expression was significantly correlated with poor patient OS (Figure 5D).

This cohort was then separated into two groups of 86 patients with low and high RIP140 expression in the corresponding tumors using the median as a cutoff value (Figure 5E,F, respectively). In this context, we observed a statistically significant association of high expression of SOX9 with an increased risk of death in CRC patients, only when their tumor exhibited low RIP140 expression (Figure 5E). Altogether, the data strongly suggested that RIP140 influences the biological activity of the SOX9 protein, in accordance with our data demonstrating a strong effect of RIP140 on SOX9 expression and transcriptional activity.

## 4. Discussion

In the present study, we demonstrated that RIP140 is a major transcriptional regulator of Sox9 expression and activity with significant consequences for PC differentiation and intestinal tumorigenesis.

We first demonstrated that the expression of RIP140 and SOX9 are inversely correlated, both in the normal mouse intestinal epithelium and in human CRC samples. This reverse correlation might reflect an opposite regulation of these two genes by the Wnt signaling pathway. Indeed, it has been known for many years that Sox9 is a positive target of the Wnt pathway [18]. On the contrary, this study identified Rip140 as a Wnt-repressed gene. Although most studies have focused on the Wnt-mediated activation of transcription, several laboratories reported data suggesting that the activation of Wnt signaling also leads to repression of gene expression [41]. Moreover, a new mode of repression of Wnt target genes has been described, in which recognition of a novel DNA element by TCF specifies that β-catenin acts as a transcriptional repressor [42]. Interestingly, this specific DNA sequence was found in the RIP140 promoter (data not shown). Moreover, in line with these data, ChIP-seq characterization of the β-catenin and TCF4 cistromes in LS180 cells identified the RIP140 as a target of TCF4/β-catenin [43].

The present study also identified RIP140 as a SOX9 interactor able to inhibit its transactivation potential. Our data mapped the interaction domain of RIP140 to the HMG box of SOX9. The SOX9 HMG domain acts by binding to specific DNA sites to activate transcription of target genes [44]. It is therefore conceivable that the interaction of RIP140 with the HMG box of SOX9 strongly impaired its binding to DNA, thus explaining the observed inhibition of transactivation and the decrease in the levels of its target genes. Although very few reports have been published, it has been demonstrated that SOX9 interacts with other transcription factors, including β-catenin [11], RUNX2 (runt related transcription factor 2) [45], NFY [46]**,** and Gli proteins [47]. Therefore, the regulation of SOX9 transcriptional activity might occur in the context of a SOX9 multiproteic complex, which might differ according to the cell and promoter contexts.

Concerning the biological consequences arising from the inhibition of Sox9 signaling, we observed a strong impact of RIP140 on PC differentiation. These cells are major regulators of the intestinal homeostasis implicated in host–microbiota interactions [6], in the sensing of environmental changes [7], and in intestinal inflammation [8]. In the small intestine, PCs are the primary source of antimicrobial proteins and peptides. A deficit in PC defensins affects the antibacterial host-defense capacity of the intestinal mucosa and is observed in ileal Crohn’s disease, a subgroup of inflammatory bowel disease, whose pathogenesis endorses a concept of an ongoing immune activation driven by normal bacterial flora [48]. Based on our results, it will be interesting to investigate whether RIP140 might play a role in this pathology. Moreover, further studies will be needed to determine if RIP140 could also play a role in the PC metaplasia associated with the detection of PC-like cells at the base of colonic crypts in inflammatory cecum and right colon [49,50]. In addition, PCs provide support to stem cells by secreting EGF, Wnt3a, and DLL4, while under injury conditions, PCs acquire stem features and generate all type of intestinal epithelial cells by activating Notch and Wnt signals [51]. Due to its negative effect on intestinal epithelial turnover after irradiation and on PC differentiation, determining whether RIP140 modulates the intestinal stem cell population could be an attractive proposition.

The oncogenic role of Sox9 [52,53] is linked to an increase in cell proliferation ([23] and Figure 5B of the present work) and in vivo tumorigenicity [23,24] and might be under the control of RIP140. Indeed, our data demonstrate that the expression level of RIP140 influences the potency of SOX9 as a predictor of patient survival. RIP140 expression could therefore be used as a marker for the identification of patients who might benefit from personalized anticancer therapies based on a SOX9 targeting by specific inhibitors or compounds that attenuate its expression [54]. Moreover, it would be interesting to investigate whether the same cross-talk between SOX9 and RIP140 also occurs in other types of cancer, including breast cancer [55].

## 5. Conclusions

This study demonstrates for the first time that the transcription coregulator RIP140 inhibits the PC lineage through the regulation of SOX9 expression and activity. In addition, we found a negative correlation between RIP140 and SOX9 expression in human CRC tissues. Interestingly, we observed a negative regulation of RIP140 expression by the Wnt pathway in CRC cells. Finally, we determined that RIP140 and SOX9 present opposite effects on cell proliferation and opposite prognosis markers in CRC. This work thus reinforces the role that RIP140 plays in intestinal tumorigenesis by controlling a major intestinal transcription factor involved in CRC.

## Data Availability

The links to publicly archived datasets analyzed in this study are available on request from the corresponding author.

## Supplementary Materials

The following are available online at https://www.mdpi.com/article/10.3390/cancers13133192/s1, Figure S1: RIP140 regulates Paneth cell lineage in mouse intestine of constitutive mouse models by inhibiting SOX9 signaling. Figure S2: Comparative effects of SOX9 wild-type and mutants. Figure S3: Effect of RIP140 and SOX9 silencing, alone or in combination, on cell proliferation. Table S1: primer sequences.
