# Supplementary material for: RIP140 Represses Intestinal Paneth Cell Differentiation and Interplays with SOX9 Signaling in Colorectal Cancer
Source: Cancers (Basel). 2021 Jun 26;13(13):3192. doi: 10.3390/cancers13133192 (PMC8268705; doi:10.3390/cancers13133192)
Supplement: Supplementary file 1 [file cancers-13-03192-s001.zip › cancers-1217454-supplementary-final.pdf]

cancers

MDPI

Article

RIP140 Represses Intestinal Paneth Cell Differentiation and Interplays with SOX9 Signaling in Colorectal Cancer

Antoine Gleizes, Mouna Triki, Sandrine Bonnet, Naomi Baccari, Gabriel Jimenez-Dominguez, Aurélie Covinhes, Nelly Pirot, Philippe Blache, Rong Yuan, Balázs Győrffy, Vincent Cavaillès and Marion Lapierre

Suppelementary materials:

1. Supplemental Experimental Procedures.

Immunofluorescence (IF) analysis. Mouse tissues were fixed with 4% paraformaldehyde, embedded in paraffin and sectioned (5µm). For immunufluorescence analyses, following incubation in citrate buffer solution, paraffin-embedded tissue sections were incubated with blocking serum for 3 h to reduce non-specific binding. Sections were then incubated with antibodies specific for SOX9. IF revelation was performed using an Alexa-conjugated secondary antibody (Invitrogen). After washing, sections were counterstained with Hoechst (Sigma Aldrich) and mounted for fluorescence microscopy. Negative controls using rabbit or mouse IgGs were performed and no staining was observed in these conditions. Quantification of staining was realized by the Axiovision software.

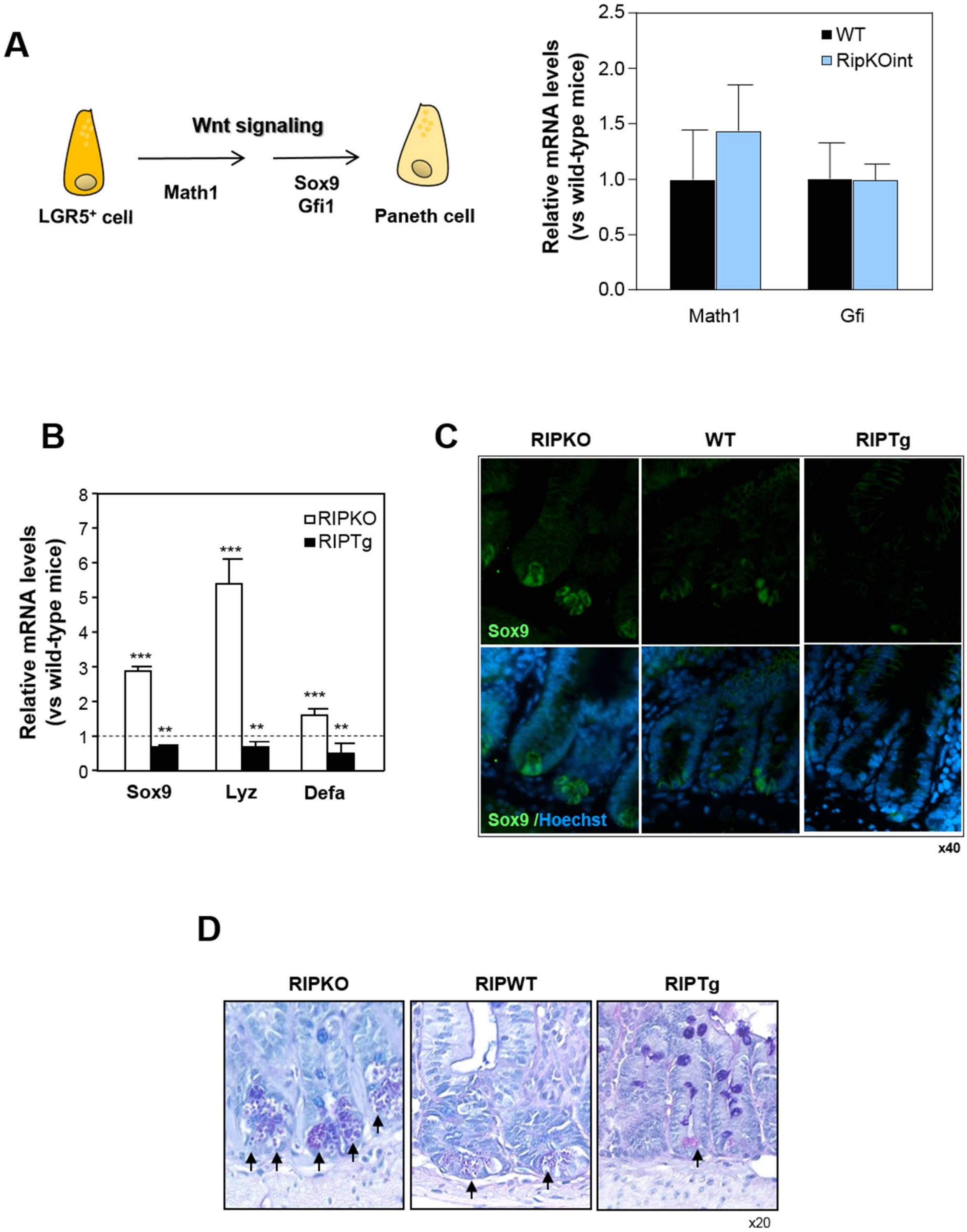

Figure S1. RIP140 regulates PC lineage and SOX9 signaling in the intestine of transgenic mouse models. A) Schematic of transcription factors involved in the differentiation of Paneth cells (left panel). RT-qPCR analysis of Math1 and Gfi1 in small intestines of WT and RipKO^{int} mice (right panel). The results are expressed in arbitrary units after normalization by RS9 mRNA levels. Values are the means ± SEM of 4 independent experiments. B) RT-qPCR analysis of Sox9, Lysozyme (Lyz) and Defensin (Defa) in small intestines from RIPKO, WT and RIPTg mice. C) Imunofluorescence of Sox9 expression in paraffin-embedded sections of small intestine from RIPKO, WT and RIPTg mice. Images show merged Sox9 detection (green) and nuclear staining (blue). Original magnification ×20. D) PAS-BA coloration of RIPKO, WT and RIPTg small intestines. Arrows indicate paneth cells. A Student t test was used for statistical analysis: *p <0.05, **p <0.001 and ***p <0.001.

.

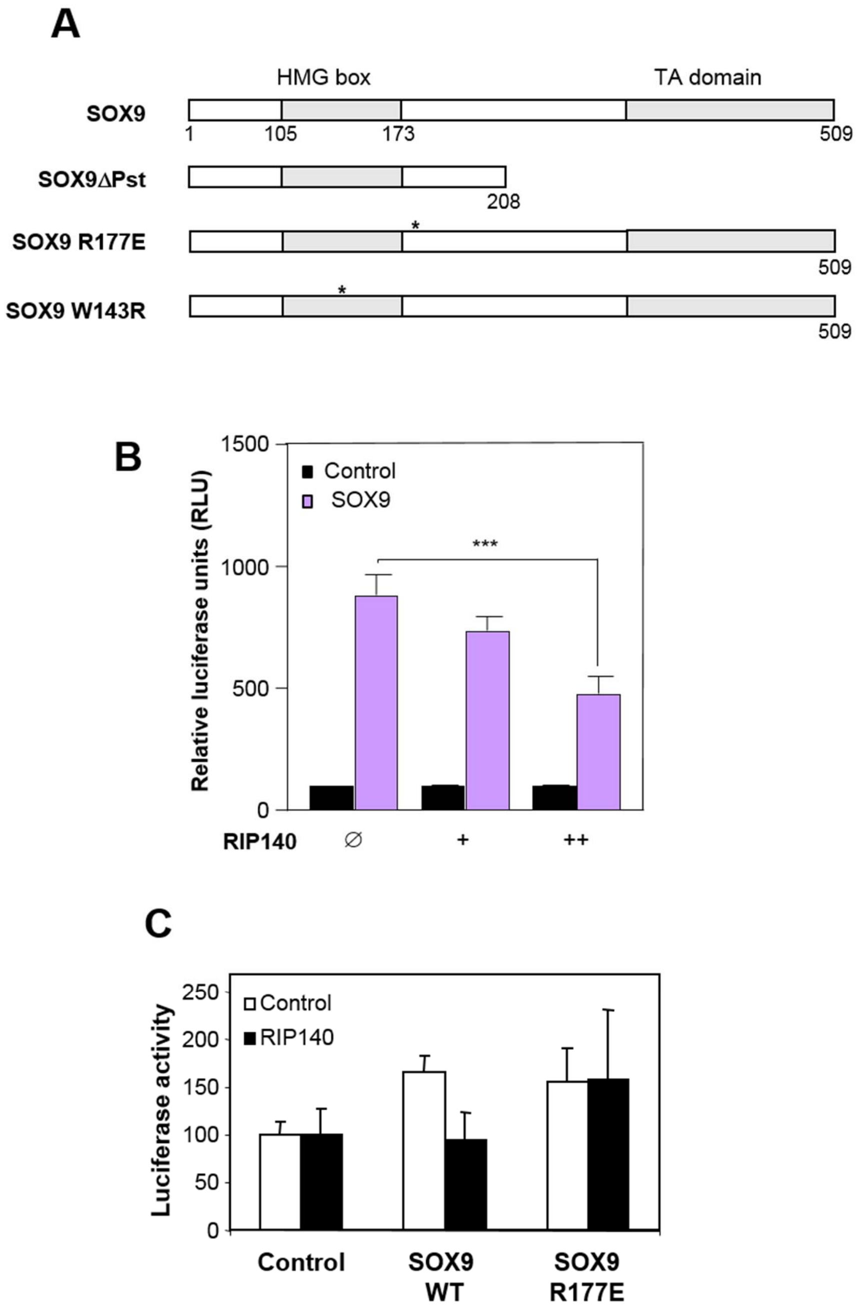

Figure S2. Comparative effets of SOX9 wild-type and mutants. A) Schematic representation of SOX9 mutants. The asterisks indicate the position of the corresponding mutation. B) HCT116 cells were transfected with SOX9 reporter vector together or not with RIP140 expression plasmid and either wild-type or mutant SOX9 expression vector. Relative luciferase activity was expressed as the mean ± S.D.; $n = 3$. C) HCT116 cells were transfected with SOX9 response element reporter vector together or not with SOX9 expression plasmid and increasing doses of RIP140 expression vector. Relative luciferase activity was expressed as the mean ± SEM.; $n = 3$. A Student t test was used for statistical analysis: *p <0.05, **p <0.001 and ***p <0.001.

.

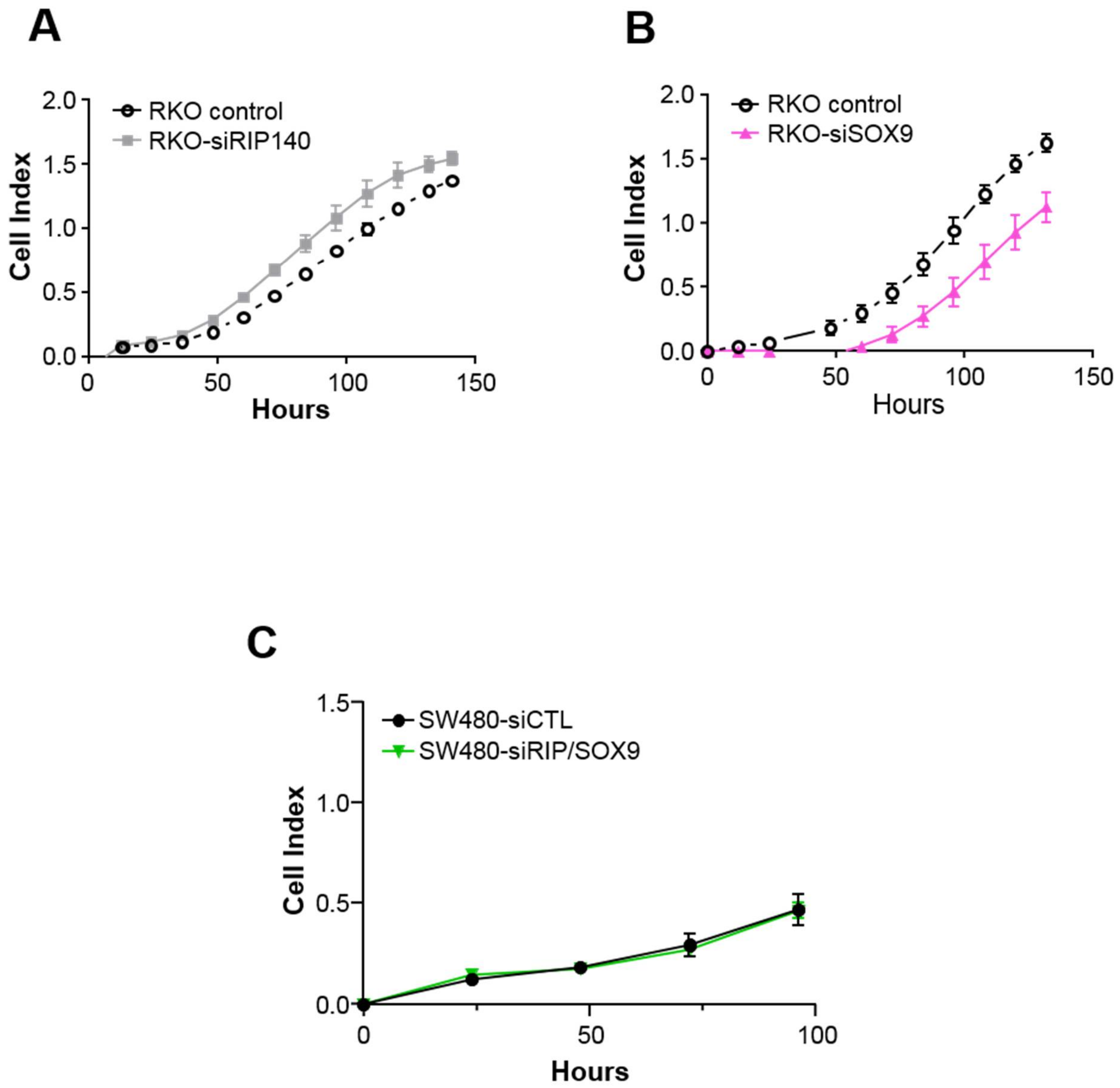

Figure S3. Comparative effets of RIP140 and SOX9 silencing on cell proliferation. Cell index corresponding to the number of RKO CRC viable cells, without (RKO-control) or with siRNA-mediated knock-down of A) RIP140 (RKO-siRIP) or B) SOX9 expression (RKO-siSOX9). C) Cell index corresponding to the number of SW480 CRC viable cells, without (RKO-control) or with siRNA-mediated knock-down of RIP140 and SOX9 expression (SW480-siRIP/SOX9).

Table S1. primer sequences.

Gene Symbol	Forward Sequence	Reverse Sequence
mDefa	GGACCAGGCTGTGTCTGTCT	TTGCAGCCTCTTGCTCTACA
mLyz	CATGGCGAGCACACTGTCA	GCGGTCAGACTCCGCAGTT
mRip140	AGAACGCACATCAGGTGGCA	GATGGCCAGACACCCCTTTG
mSox9	TCGGTGAAGAACGGACAAGC	TGAGATTGCCCAGAGTGCTCG
hCTNNB	GCTGGGACCTTGCATAACCTT	ATTTTCACCAGGGCAGGAATG
hRIP140	AATGTGCACTTGAGCCATGATG	TCGGACACTGGTAAGGCAGG
hSOX9	CGGAGGAAGTCGGTGAAG	CTGGGATTGCCCCGAGTGCT

Copyright: © 2021 by the authors. Licensee MDPI, Basel, Switzerland. This article is an open access article distributed under the terms and conditions of the Creative Commons Attribution (CC BY) license (http://creativecommons.org/licenses/by/4.0/).
